# Supplementary material for: Effect of Low Light Stress on Distribution of Auxin (Indole-3-acetic Acid) between Shoot and Roots and Development of Lateral Roots in Barley Plants
Source: Biology (Basel). 2023 May 29;12(6):787. doi: 10.3390/biology12060787 (PMC10295069; doi:10.3390/biology12060787)
Supplement: Supplementary file 1 [file biology-12-00787-s001.zip › biology-2387873-supplementary.pdf]

Supplementary Materials

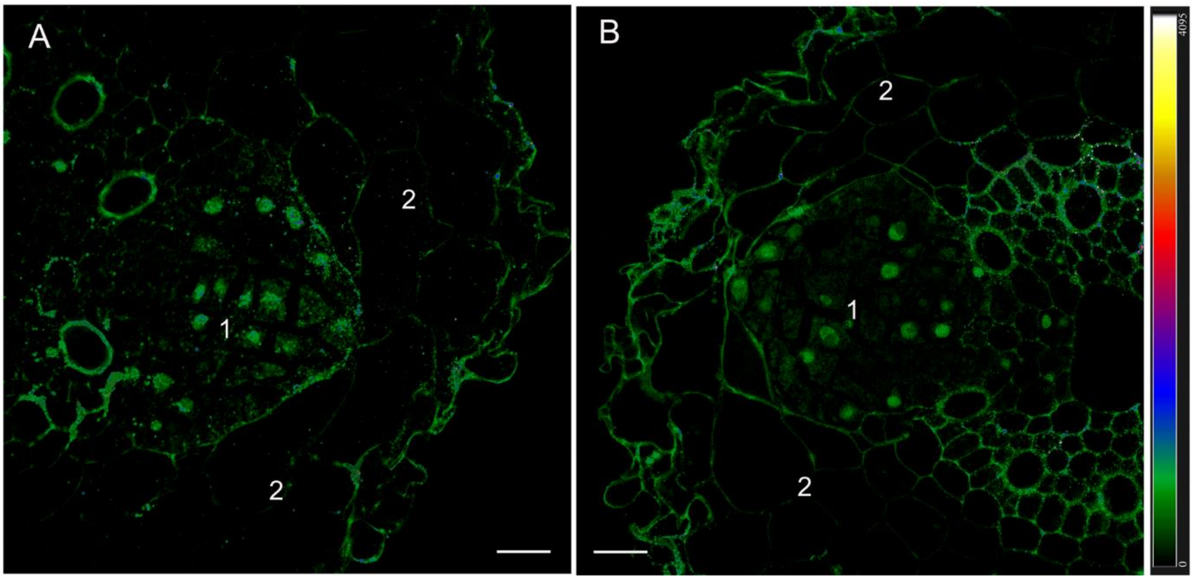

Figure S1. Immunohistochemical localization of IAA in cross sections of the roots of barley plants after their growing during one day at different illumination levels. Images were taken from 10 independent sections per treatment and figure shows representative images. Intensity of fluorescence is displayed as a color-coded heatmap. (**A**)—control (165 µmol m^{-2} s^{-1}), (**B**)—reduced illumination (45 µmol m^{-2} s^{-1}). Scale bar 20 µm, 1 – primordia of lateral roots, 2 – root cortex.
